# 245 MHz bandwidth organic light-emitting diodes used in a gigabit optical wireless data link

**DOI:** 10.1038/s41467-020-14880-2

**Published:** 2020-03-03

**Authors:** Kou Yoshida, Pavlos P. Manousiadis, Rui Bian, Zhe Chen, Caroline Murawski, Malte C. Gather, Harald Haas, Graham A. Turnbull, Ifor D. W. Samuel

**Affiliations:** 1 Organic Semiconductor Centre, SUPA, School of Physics and Astronomy, University of St Andrews, St Andrews, KY16 9SS UK; 2 Li-Fi R&D Centre, Institute for Digital Communications, University of Edinburgh, Edinburgh, EH9 3JL UK; 3 Present Address: Kurt-Schwabe-Institut für Mess- und Sensortechnik e.V. Meinsberg, Kurt-Schwabe-Str. 4, 04736 Waldheim, Germany

**Keywords:** Organic LEDs, Photonic devices

## Abstract

Organic optoelectronic devices combine high-performance, simple fabrication and distinctive form factors. They are widely integrated in smart devices and wearables as flexible, high pixel density organic light emitting diode (OLED) displays, and may be scaled to large area by roll-to-roll printing for lightweight solar power systems. Exceptionally thin and flexible organic devices may enable future integrated bioelectronics and security features. However, as a result of their low charge mobility, these are generally thought to be slow devices with microsecond response times, thereby limiting their full scope of potential applications. By investigating the factors limiting their bandwidth and overcoming them, we demonstrate here exceptionally fast OLEDs with bandwidths in the hundreds of MHz range. This opens up a wide range of potential applications in spectroscopy, communications, sensing and optical ranging. As an illustration of this, we have demonstrated visible light communication using OLEDs with data rates exceeding 1 gigabit per second.

## Abstract (web summary)

Organic LEDs (OLEDs) have generally been considered to be slow devices. Through engineering the structure and materials of OLEDs, the authors achieve a breakthrough in the high-speed operation of OLEDs and demonstrate a 1 Gbps optical wireless link using the OLEDs.

## Introduction

Organic semiconductors are now well established as a thin-film electronics platform for displays, solar power and printed electronics^1–4^. Their optical and electronic properties can be tailored through molecular design to give very efficient light emission, and to tune the band gap energy or thin film morphology^5–7^. Organic electronics and organic light emitting diodes (OLEDs) in particular are now widely adopted in smart devices and wearables^8–11^. They can be produced in very high volumes on very thin, flexible plastic substrates, at low cost^12,13^, with high density integration of pixels of different materials or for very large area electronics. While high power efficiency has been achieved for OLEDs, organic solar cells, and photodiodes, their temporal performance is generally thought to be intrinsically slow. For example, in comparison to inorganic LEDs, OLEDs have low modulation bandwidth which is mainly attributed to their low charge mobility and high capacitance. The charge mobility of organic semiconductor materials (especially the ones used in OLEDs) is several orders of magnitude lower than for inorganic semiconductors^14^. In addition, OLEDs have a planar structure of several tens to hundreds of nanometers thickness. This forms a high capacitance which limits bandwidth drastically^14,15^, with few reports exceeding 10 MHz^16^. A striking consequence of this is the highest reported data rate for an OLED optical data link is 51.6 Mbps by Chen et al.^17^, whereas GaN LEDs have achieved visible light communication (VLC) data links of several Gbps^18–20^.

OLEDs with high electrical bandwidth (of hundreds of MHz or above) could enable a wide range of new applications of organic electronics. For example, OLEDs are an attractive light source for low cost integration in lab-on-a-chip formats for disposable point of care diagnostics^21^; fast intensity modulation would enable time-resolved fluorescence measurements of biomarkers^22^, broadening the scope for diagnostic tests with high specificity. Visible light communications is a promising approach to address the rapidly increasing global demand for wireless communications bandwidth^23–25^; fast OLED transmitters could play a role in data links with smart phones, smart label sensors or secure contactless communications between credit cards and ATMs. Fast modulation of OLED arrays and displays could also enable new approaches to fluorescence lifetime imaging^26–28^, optical detection^29^ and ranging^30,31^, and structured light imaging^32,33^.

Here, we report a significant breakthrough in speeds of OLEDs. To achieve this, we carefully investigated the factors limiting the bandwidth of OLEDs and how to overcome them. We explored the effect of active area size, electrode design, and the emitting material. By simultaneously optimizing all three of these aspects, we fabricated OLEDs that show a significant improvement in bandwidth. We illustrate one area of possible application of these very fast OLEDs by showing that they can be used as a transmitter for a free space communication link at a rate of 1.13 Gbps over a distance of 2 m. To the best of our knowledge, this constitutes an improvement of a factor of 20 over previously reported OLED VLC data rates^17^, and is achieved over a much larger distance. We believe that these results will pave the way for efficient, low-cost, and high-speed organic optoelectronics, with potential applications in secure communications, point of care diagnostics, and optical imaging and ranging.

## Results

### Overview

Three generations of OLED devices were developed and a combination of optoelectronic and photophysical measurements made to identify the key factors limiting their modulation bandwidth and overcome them. Each OLED generation was then characterized in a data transmission measurement to assess their capacity to usefully exploit their modulation bandwidth. An orthogonal frequency division multiplexing (OFDM) modulation scheme was used, which permits adaptive allocation of data and energy in different frequency bands to optimally utilize the available bandwidth (see Methods section for further details)^18,34,35^.

### First generation OLEDs with p-i-n structure and fluorescent emitter

**Fig. 1:**
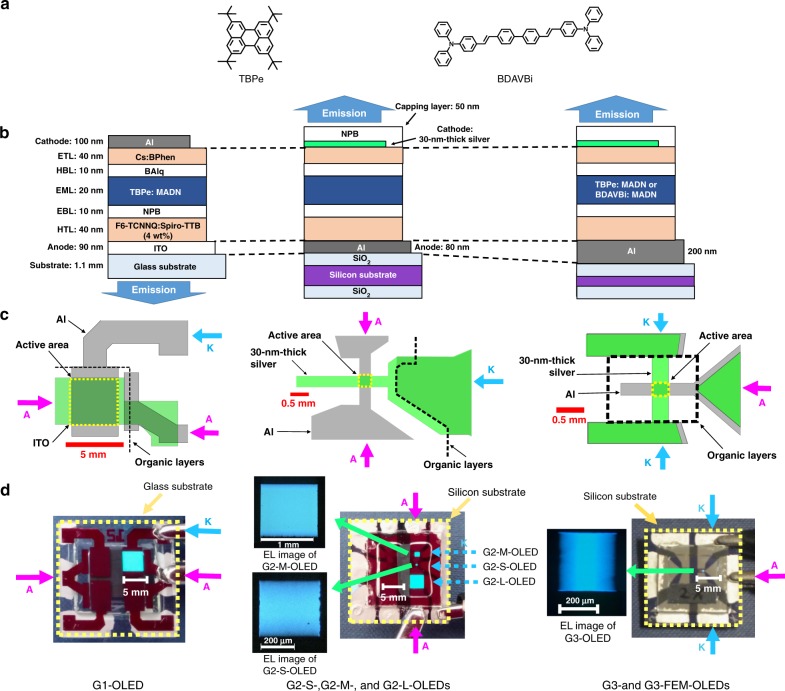
Summary of the three generations of OLEDs investigated. **a** Chemical structure of the emitter molecules used. **b** Cross section of OLEDs. ETL electron transport layer, HTL hole transport layer, EML emission layer, HBL hole blocking layer, EBL electron blocking layer. **c** Schematic illustrations of the top view of the OLEDs. Purple arrows indicate the contacts for anode (A) and blue arrows indicate the contacts for cathode (K). **d** Photographs of the OLEDs and corresponding images under a microscope of devices emitting blue light.

The first generation of OLEDs were designed with a p-i-n structure^36^ with doped charge transport layers, based on an established blue fluorescent emitter, 2,5,8,11-tetra-*tert*-butylperylene (TBPe)^37–39^ (hereafter termed “G1-OLEDs”, see Fig. 1 for details). This fluorescent emitter was chosen over more efficient phosphorescent and thermally activated delayed fluorescent emitters due to its substantially shorter luminescence lifetime^40^ (4.4 ns for TBPe doped into 2-methyl-9,10-bis(naphthalen-2-yl)anthracene (MADN) (hereafter termed “TBPe film”, see Supplementary Fig. 1a) A p-i-n structure with doped transport layers^36^ was selected to achieve good charge injection from the contacts and high conductivity. This reduced heating of the device and hence enabled operation at high current and high brightness (Supplementary Fig. 2a).

**Fig. 2:**
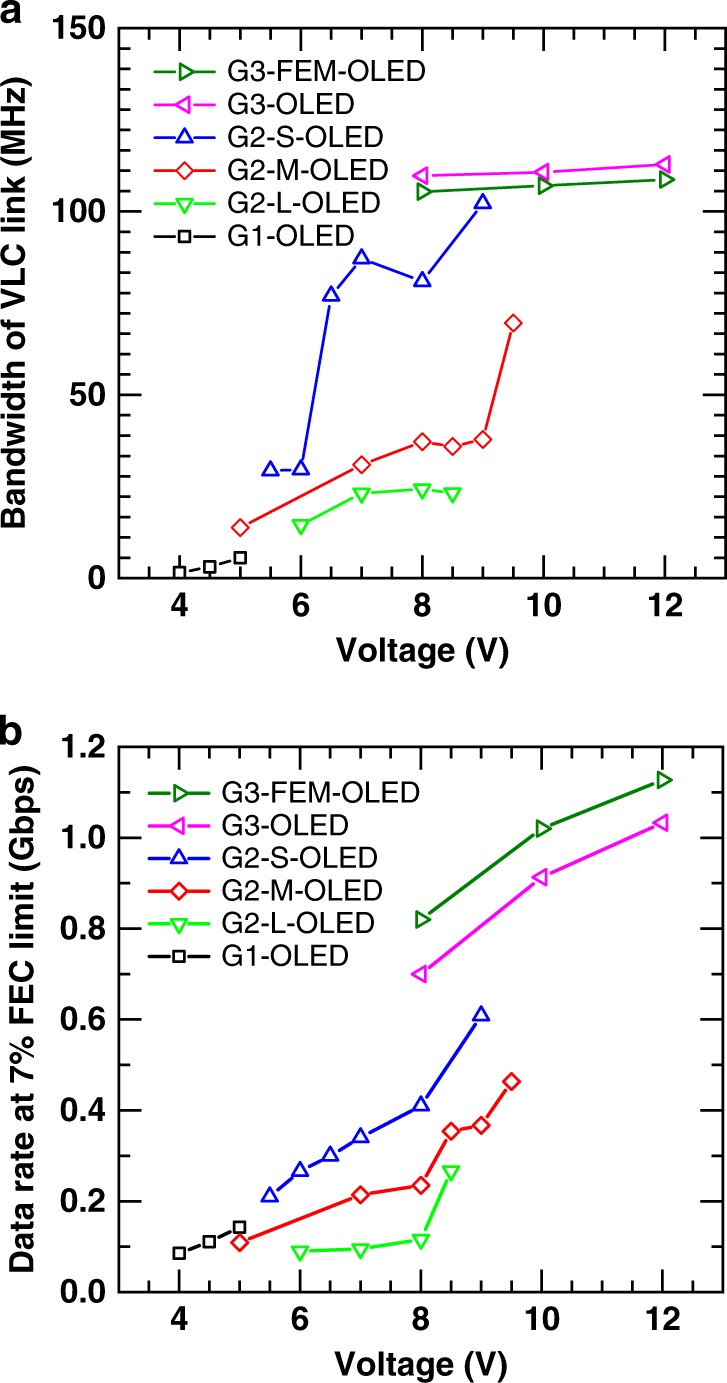
Communication performance of the OLED devices in a VLC link. **a** Bandwidth of the VLC link, and **b** data rate at 7% FEC limit for each type of OLED.

To assess the bandwidth of the G1-OLEDs, the frequency response of the system was determined (see Methods section). The overall optical link exhibits a low-pass characteristic whose bandwidth is significantly determined by the OLED. Figure 2a displays the bandwidth of the link (the frequency at which the power gain of a VLC link, [*H*]^2^, decreases 6 dB from its maximum value) as a function of the voltage applied to the G1-OLEDs. We observed an increase of the bandwidth with increasing voltage. This may be attributed to a reduction in device resistance as the voltage was increased, leading to a reduction of the electrical time constant of the OLEDs^41^ (see Supplementary Note 2). We note that the maximum voltage for the G1-OLED was limited to around 5 V by device breakdown. The details and the limiting factors of the G1-OLEDs are discussed further in the next section.

A free-space data link using the G1-OLED as transmitter was next evaluated by locating a receiver 2 m away and driving the OLED with a data stream using an implementation of direct current (DC)-biased optical orthogonal frequency division multiplexing (DCO-OFDM) (see Supplementary Note 3 for the details of data transmission optimization). Figure 2b shows the data rate at a bit error ratio (BER) of 3.8 × 10^−3^, which corresponds to the 7% forward error correction limit (7% FEC limit^42^) as a function of voltage for the G1-OLEDs. A maximum data rate of 140 Mbps (average of 4 OLED samples, Supplementary Table 1) was achieved for an applied DC voltage of 5 V.

### Second generation OLEDs of different active area with thermal management and top emission geometry

To further improve the OLED bandwidth, it is necessary to reduce the electrical time constant. To achieve this, the device design was modified to reduce both the wiring resistance, which is the resistance between the contact pads and the active area of the OLED, and the capacitance of the OLED. These steps reduced the RC time constant, and also enabled the device to operate at higher voltage. The indium tin oxide (ITO) layer (90 nm; 42 Ω/sq.^43^) was replaced with a thin silver layer (30 nm; <3 Ω/sq.), thereby reducing the resistance of the transparent electrode. Smaller OLED areas can achieve higher bandwidth^44^ due to reduced capacitance, but this comes with a penalty of lower light output, potentially limiting the signal to noise ratio (SNR) of a data link. We therefore fabricated an improved set of OLEDs with the same organic stack but in a range of different sizes to test this trade-off and find an optimized area to maximize the communication performance. We used thermal evaporation of crossed electrodes to define the device area rather than introducing insulators with photolithography, which would cause parasitic capacitance^45^. Finally, we note that for the first generation OLEDs, both their bandwidth and data rate increased with applied voltage, but were limited to a maximum value by the temperature rise of the device, which led to device breakdown^46^. To suppress this temperature rise, the second generation OLEDs were fabricated on silicon substrates, which offer high thermal conductivity^47,48^. These second generation OLEDs (hereafter termed “G2-OLEDs”) were fabricated with three different device areas: G2-S-OLEDs (1.2 × 10^−3^ cm^2^), G2-M-OLEDs (1.1 × 10^−2^ cm^2^), and G2-L-OLEDs (9 × 10^−2^ cm^2^).

Figure 2a shows the results of bandwidth measurements of a link using G2-OLEDs as a function of voltage. The bandwidth is higher for G2-OLEDs with smaller device area—for example at 8 V the bandwidths of G2-L-OLED, G2-M-OLED, and G2-S-OLED are 24, 37, and 81 MHz, respectively. It can also be seen that the bandwidth increases with increasing voltage. Both observations are substantially due to a reduction in electrical time constant. This arises from the change in device area and (as diodes have nonlinear current–voltage characteristics) a reduction in resistance as voltage is increased. The results show that reducing device size and increasing operating voltage are helpful to realize high bandwidth. The data rate of these devices is shown in Fig. 2b. For all second generation OLEDs, the data rate increases with voltage, with the G2-S-OLEDs giving the highest data rate of 610 Mbps at 9 V (average of three OLED samples).

### Third generation OLEDs with improved device configuration and faster emitter

The potential ways of increasing the modulation bandwidth of the G2-S-OLEDs are to reduce further the electrical time constant, decrease the charge transit time, and shorten the emission lifetime. These points were addressed in the third generation OLEDs (G3-OLEDs). The electrical time constant could be reduced further by reducing the device area, increasing the number of contact pads for the more resistive transparent electrode (as resistance decreases by connecting two resistors in parallel), and by increasing the thickness of the aluminum (Al) bottom electrode to reduce wiring resistance. G3-OLEDs were therefore designed with similar stack structure to the G2-OLEDs, but with the device area slightly reduced to 9.2 × 10^−4^ cm^2^, and a different electrode configuration. The cathode was re-designed with two contact pads, while the thickness of the Al anode was increased from 80 to 200 nm (see Fig. 1b).

Since the bandwidth of the G2-S-OLED is comparable to the emission lifetime of the TBPe film, OLEDs with an alternative emitter of shorter photoluminescence (PL) lifetime, 4,4′-bis[4-(diphenylamino)styryl]biphenyl (BDAVBi),^49^ were fabricated as well (G3-“fast-emission-molecule” (FEM)-OLEDs). The lifetime of BDAVBi films doped into MADN was measured to be 1.1 ns (see Supplementary Fig. 1a).

**Fig. 3:**
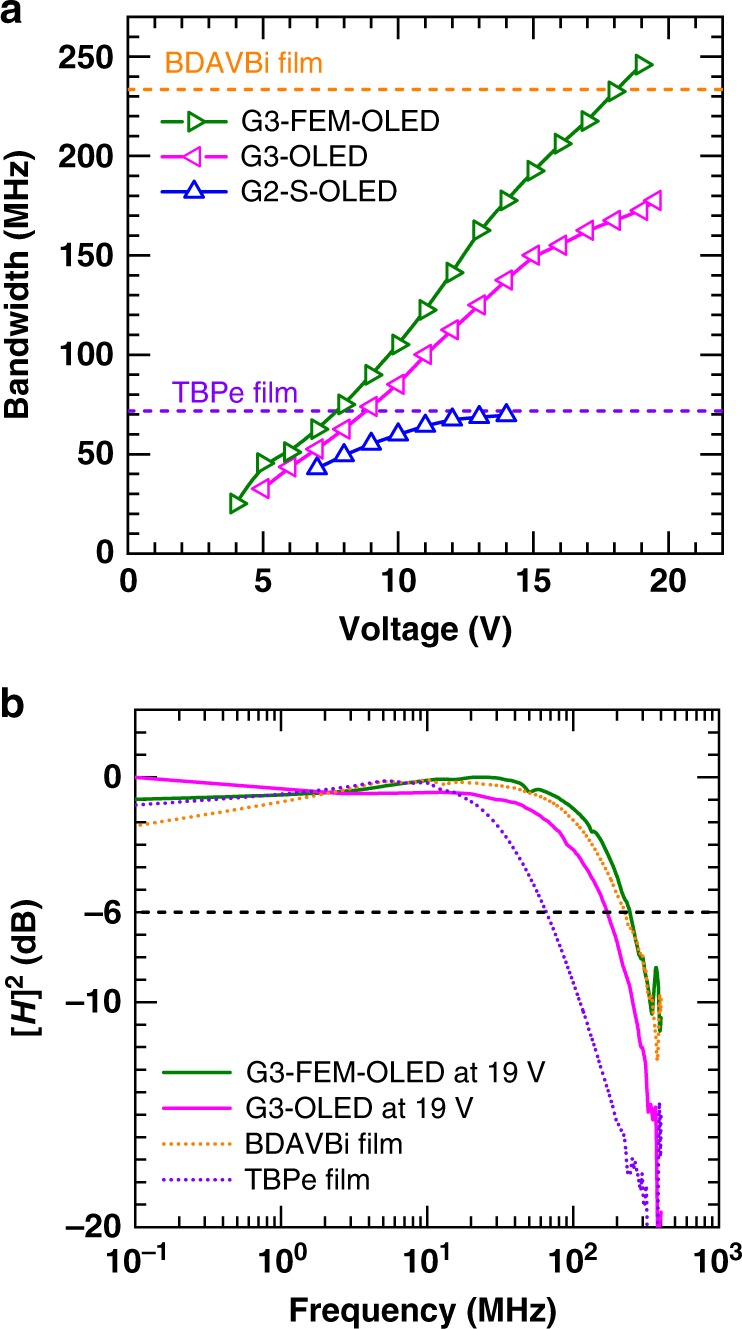
Frequency characteristics of OLED devices. **a** Bandwidth as a function of voltage for the three fastest OLED designs, and comparison with the bandwidth of the films (dashed lines). **b** Frequency response of the OLEDs at 19 V and the films under optical excitation. The black dashed line shows a channel gain [*H*]^2^ of −6 dB.

Figure 2a shows our initial measurements of the link’s bandwidth with the G3-OLED and G3-FEM-OLEDs as a function of applied voltage. Higher bandwidth was observed with the G3-OLED than with the G2-S-OLED. The bandwidth of the fastest OLEDs was then studied in more detail using a photodiode with flatter frequency response (Thorlabs, APD430A2/M)^50^ and the results are shown in Fig. 3a. The G3-OLEDs show higher bandwidth than the G2-OLEDs and the bandwidth increases substantially with increasing voltage. The G3-OLED and G3-FEM-OLEDs achieved maximum bandwidths of 178 and 245 MHz respectively, and the bandwidth of G3-FEM-OLED increases more steeply than the G3-OLED with voltage. Figure 3b shows a comparison of the frequency response of the OLEDs and the corresponding films of their emission layers. The BDAVBi film has much higher bandwidth than the TBPe film because of its shorter PL lifetime. Interestingly, it was found that the bandwidth of these OLEDs can be higher than the PL bandwidth of the emitting molecules, especially for the G3-OLED which uses TBPe as an emitter. This suggests there are additional processes in the device, such as singlet-triplet annihilation^38^, which shorten the emission lifetime.

There are two main factors that contribute to the increase of bandwidth with increasing voltage. One is that the higher electric field leads to a shorter charge transit time. The other is that the reduction in resistance reduces the electrical time constant. In the G3-FEM-OLED the resistance of the device is large compared with the wiring resistance at all operating voltages. In this scenario, the effect of the change in device resistance is minor^41^, and the main effect is the reduction of charge transit time. In combination with the short emission lifetime, this leads to the very high bandwidth for an organic semiconductor device of 245 MHz. We note that this measurement was made with a detector with a flat frequency response up to 400 MHz whereas the VLC link used a detector with a resonance around 100 MHz (Supplementary Fig. 6).

Finally, the VLC performance of the G3- and G3-FEM-OLEDs was investigated. Figure 2b and Supplementary Table 1 show the data rate at the 7% FEC limit of the OLEDs in a 2 m data link. Both G3-OLEDs achieved data rates exceeding 1 Gbps. The highest data rate was for the G3-FEM-OLED which exhibited a maximum data rate of 1.13 Gbps (average of 3 OLED samples) for a peak to peak voltage of 1.3 V with DC offset of 12 V and mudulation bandwidth of 165 MHz.

**Fig. 4:**
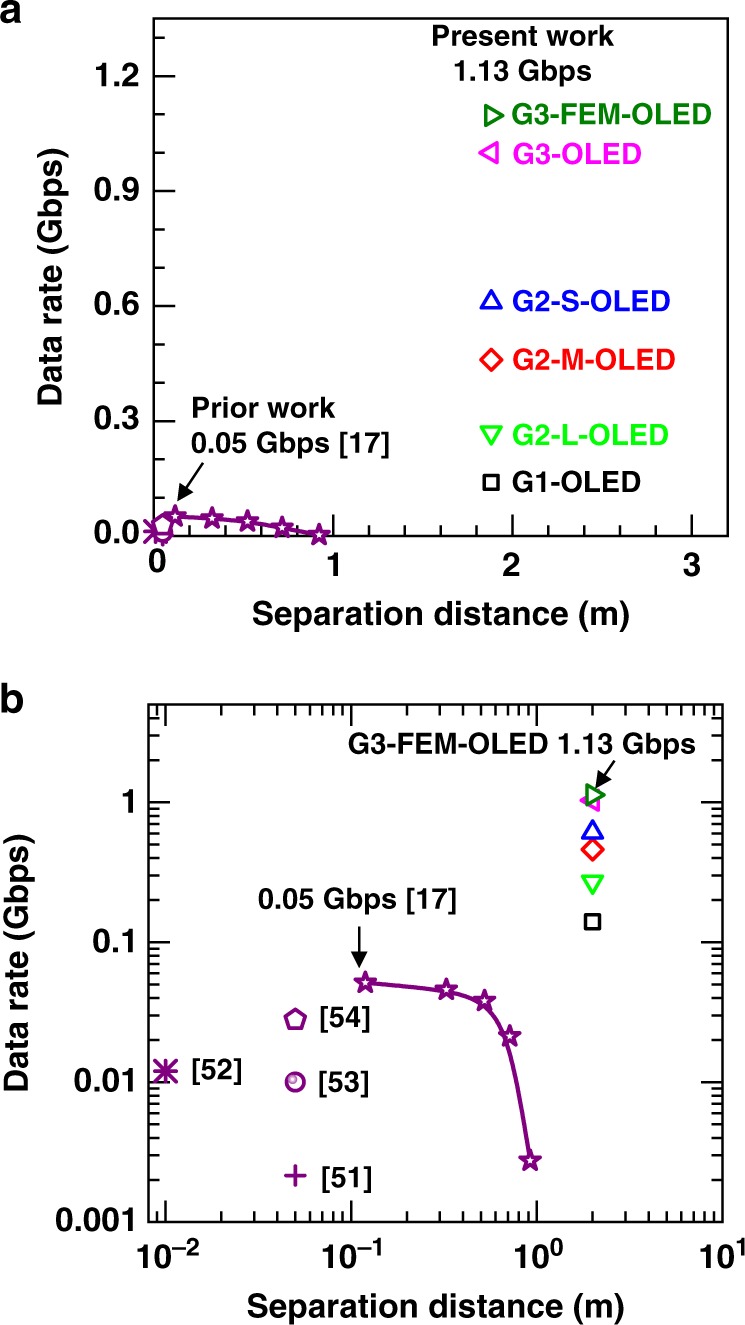
Comparison of reported OLED data link speeds. Data rate of our OLEDs compared to literature devices as a function of separation distance between OLED and receiver in a wireless data link: **a** on a linear scale and **b** on a logarithmic scale.

Like other OLED-VLC work we used a lens at the transmitter and receiver, and this allows us to compare our results with literature reports in Fig. 4. Our data rate of 1.13 Gbps with the G3-FEM-OLED is 22 times greater than that of earlier devices^17,51–54^. Furthuremore, while Chen et al. recently demonstrated that the data transmission rate significantly drops with increasing separation distance^17^, we achieved our value in a link of a practical length of 2 m, i.e., 16 times longer than they used.

## Discussion

We have demonstrated a breakthrough in high speed OLED performance. Organic optoelectronic devices are usually thought to be slow, but we have shown how the potential limitations of electrical time constant, low mobility and excited state lifetime can be overcome by careful device design and materials selection. This paves the way to a new generation of organic electronic and optoelectronic devices working much faster than previously thought possible. We have illustrated this by making a 20-fold advance in the VLC performance to achieve data rates of 1.13 Gbps from OLEDs as data transmitter over a 2 m link. While the lens-coupled 2 m data link demonstrated here requires point-to-point alignment of the transmitter and receiver, the fast OLEDs could potentially be applied lens-free in shorter contactless datalinks, or with high gain alignment-tolerant receivers^55–57^. Moreover, our results suggest that OLEDs may be suitable for a range of potential applications where fast modulation is required, spanning secure communications, point of care diagnostics, and optical imaging and ranging. The principles developed here may also prove useful for making other organic electronic devices faster than previously thought possible.

## Methods

### Fabrication of OLEDs and films of emission layers of OLEDs

The OLEDs were fabricated as follows: metals and organic materials were thermally evaporated through a shadow mask in a vacuum chamber at a base pressure of 10^−7^ mbar (Angstrom Engineering Inc., EvoVac). The OLED layer stack consisted of 40 nm 2,2′,7,7′-tetrakis(*N,N*′-di-*p*-methylphenylamino)-9,9′-spirobifluorene (Spiro-TTB) p-doped with 2,2′- (perfluoronaphthalene-2,6-diylidene)dimalononitrile (F6-TCNNQ) (4 wt%) as a hole transport layer (HTL), 10 nm *N,N*′-di(naphtalene-1-yl)-*N,N*′-diphenylbenzidine (NPB) as an electron blocking layer (EBL), 20 nm emission layer (EML, detailed below), 10 nm bis-(2-methyl-8-quinolinolato)-(4-phenyl-phenolato)-aluminum (III) (BAlq) as a hole blocking layer (HBL), and 40 nm cesium-doped 4,7-diphenyl-1,10-phenanthroline (BPhen) as an n-doped electron transport layer (ETL). Calibrated quartz crystal microbalances were used to control deposition rates and the final thicknesses of each layer. All materials were purchased from commercial suppliers and used without further purification. All OLEDs were encapsulated under nitrogen atmosphere using 1.1 mm thick glass lids (Shanghai Amerina Optoelectronic Co., Ltd.), epoxy resin (Norland Products Inc., Norland Optical Adhesive 68), and an additional moisture getter (Dynic Corporation, HD-071210T-50S). A similar procedure was used to make films of the emission layers of the OLEDs although films for PL quantum yield were not encapsulated.

The G1-OLED was fabricated onto 1.1 mm thick glass substrates coated with a 90 nm thick pre-patterned indium tin oxide (ITO) anode (Thin Film Devices Inc.). The device stack was capped with a 100 nm thick aluminum cathode. The EML consisted of the fluorescent emitter 2,5,8,11-tetra-*tert*-butylperylene (TBPe), which was doped at 1.5 wt% in the host 2-methyl-9,10-bis(naphthalen-2-yl)anthracene (MADN). The active area of these G1-OLEDs was 16.1 mm^2^.

The G2- and G3-OLEDs were fabricated on silicon substrates with a thickness of 675 µm coated with a 300 nm silicon dioxide (SiO_2_) layer on both sides to prevent electrical leakage through the substrate. Aluminum anodes were evaporated onto the SiO_2_ layer through a shadow mask with different aperture sizes. The organic material stack was subsequently evaporated as detailed above. The EML of the G2- and G3-OLEDs consisted of TBPe, which was doped at 1.5 wt% in MADN. The EML of G3-FEM-OLEDs consisted of 4,4-bis[4-(diphenylamino)styryl]biphenyl (BDAVBi) doped at 3 wt% in MADN. A 30 nm silver layer was evaporated on top as a semi-transparent cathode and the stack was finished with a 50 nm thick NPB capping layer. Shadow masks were changed without breaking vacuum. A single layer of aluminum was used as a bottom electrode instead of the commonly used multi-layer of aluminum and silver^58,59^ as this was found to give higher device fabrication yield and more stable operation. The active areas were measured from electroluminescence (EL) images of the operating OLEDs under a microscope (ZEISS, Axio Lab A1, (see Fig. 1d)).

### OLED characterization

Current density-voltage-radiance characteristics were measured using a source meter (Keithley Instruments, Inc., 2400 SourceMeter), and a multimeter (Keithley Instruments, Inc., 2000 Multimeter) with a calibrated Si photodiode. Emission spectra from the OLEDs under constant current operation at ≈6 mA/cm^2^ were recorded by a CCD spectrograph (Andor Technology Ltd., DV420-BV), and used to calculate the radiance. The external EL quantum efficiency (EQE) of the OLEDs was estimated assuming a Lambertian emission pattern.

The frequency response of G2-S-, G3-, and G3-FEM-OLEDs was measured using a photodiode with a linear frequency response (Thorlabs, APD430A2/M, −3 dB bandwidth at 400 MHz). The OLEDs and the receiver were connected to a network analyser (Keysight, E5061B) and the measurement known as “S21” measurement was conducted to estimate the frequency response of the OLEDs at different DC bias voltages and at frequencies in the range 0.1–500 MHz. Results are shown up to 400 MHz due to low SNR above this frequency. The frequency response of films of the emission layers were measured in a similar way but with excitation by a laser at 405 nm (US-Lasers Inc., D405-20) connected to the network analyser through a diode driver (Thorlabs, LDC210C) and with a long-pass filter (Comar Optics Ltd, 510 IY 50) inserted between the lenses. The frequency response of the laser was measured and the frequency response of the films was estimated by dividing the measured frequency response of the films by the frequency response of the laser.

### VLC link set-up

In all the data transmission experiments, orthogonal frequency division multiplexing (OFDM) was used. It is a well-known and widely applied modulation scheme in visible light communication^18,34,35^. OFDM was selected because it is adaptive to communication systems properties, and permits adaptive allocation of data and energy at different frequency bands. It further offers advantages such as cost-effective equalization with single-tap equalizers and an easy way to avoid low-frequency interference cause by ambient light.

Supplementary Fig. 8 shows a schematic illustration of the set-up for VLC measurement. The OLEDs were mounted in tailor-made device holders. All communication experiments were conducted under typical office illumination levels. This included natural light from windows in combination with artificial light from fluorescent lamps. The link followed a typical setup for VLC experiments^60,61^. For the G2-, G3- and G3-FEM-OLEDs, a heat sink with a fan was used to dissipate Joule heating at high driving powers. The modulated digital signal was passed onto an arbitrary waveform generator (AWG, Agilent, 81180A) with analog output. The maximum peak-to-peak voltage (*V*_pp_) available from the AWG was 2 V so a broadband amplifier (Mini-Circuits, ZHL-6A-S+) was used for higher voltages. To measure the *V*_PP_ produced by the amplifier, we applied a 120 MHz sine wave with the same *V*_PP_ from the AWG as during VLC measurement to the amplifier. The output voltage of the amplifier was then measured using a bias tee (Mini-Circuits, ZFBT-4R2GW) and oscilloscope (Agilent, MSO7104B). *V*_PP_ was adjusted to maximize data rate at each bias voltage. During VLC measurement, the signal created by the AWG was combined with a DC bias voltage (*V*_DC_) from a power supply (Keithley Instruments, Inc., 2400 SourceMeter) through the bias tee and used to drive the OLEDs. Aspheric condenser lenses (Thorlabs, ACL50832U-A) with numerical aperture of 0.76 and focal length of 32 mm were used to collimate and focus the light onto the receiver circuit. The length of the communications link (distance between the lenses) was 2 m. The custom-made receiver circuit for the OLEDs comprised a silicon avalanche photodiode (Hamamatsu, S8664-10K) with an active area of 0.78 mm^2^ and the cutoff-frequency of 530 MHz and a transimpedance amplifier with an operational amplifier (Texas Instruments, OPA847). The output from the receiver circuit was captured by the oscilloscope through a low pass filter (Mini-Circuits, SLP-150+), and then processed using a laptop computer. The VLC link used an implementation of DCO-OFDM. An adaptive bit loading and power loading algorithm based on the work by Campello^62^ was used to achieve optimized experimental results (Details are given in supporting information). All the digital signal processing was implemented in numerical calculation software (MathWorks, MATLAB®).

### OLED characterization for communications

In order to estimate the frequency response of the end-to-end system, including OLEDs, multiple frames of known quadrature phase shift keying (QPSK) modulated DCO-OFDM signals were sent through the link. In each frame transmission, the channel gain (*H*) was measured at each subcarrier and then averaged over several OFDM frames. After estimating the channel gain at each subcarrier, the average noise power, $\sigma _n^2$, was measured as the difference between the received power (*P*_Rx_), i.e., signal plus noise power and the noise-free received signal power, *H*^2^*P*_Tx_, i.e., the transmitted power scaled by the estimated channel gain. Finally, the SNR was estimated at each subcarrier as the ratio between the received signal power and noise power (i.e., ${\mathrm{SNR}} = H^2P_{{\mathrm{Tx}}}/\sigma _n^2$). The bandwidth estimated for OLEDs is the frequency at which the value of [*H*]^2^ decreases 6 dB from the maximum value of [*H*]^2^.We confirmed that data transmission was due to the light emitted from the OLEDs by comparing the SNR before and after inserting a piece of paper to block the light in a shorter VLC link of 5 cm (see Supplementary Fig. 9). It was found that the SNR drops to almost 0 dB when the light is blocked.

Supplementary Figs. 10–13 show the measured BER as a function of data rate for each generation of OLEDs. The data rates are compared at a BER of 3.8 × 10^−3^, which corresponds to the 7% forward error correction (FEC) limit. This was done by linear interpolation between the nearest higher and lower data points. A few OLEDs of each type were measured. The results for every device are shown Supplementary Table 1, together with their average.

### Characterization of films of emission layers of OLEDs

Steady state photoluminescence (PL) spectra of the films were recorded in a fluorimeter (Edinburgh Instruments, FLS980). Transient PL decay curves were recorded on the same fluorimeter by time-correlated-single-photon-counting (TCSPC). Excitation was by a 379 nm laser diode (Pico Quant, LDH-D-C-375) operating at a repetition rate of 300 kHz. The measured PL decay curves were then fitted by a two exponential decay model considering the instruments response function (IRF) and an averaged emission lifetime (<*τ*>) of the films was estimated using the following equation; $< \tau > = \gamma _1\tau _1 + \gamma _2\tau _2$, where *τ*_1_ and *τ*_2_ are the emission lifetime of each component and *γ*_1_ and *γ*_1_ are the contribution of the emission from each component to the total emission (i.e., $\gamma _1 = \frac{{A_1\tau _1}}{{A_1\tau _1 + A_2\tau _2}},\gamma _2 = \frac{{A_2\tau _2}}{{A_1\tau _1 + A_2\tau _2}}$, where *A*_1_ and *A*_2_ are the pre-exponential-factors of each component). PL quantum yield of the films were measured on unencapsulated films using an integrating sphere based measurement system (Hamamatsu, C9920-02) under nitrogen flow.

## Supplementary information

Supplementary information

## Data Availability

Research data supporting this publication is available at 10.17630/2534301d-c099-4ed2-84a3-68baf0762edc. The source data underlying Figs. 2–4 and Supplementary Figs. 1–5, 6a, 7, 9b, 10, 12, and 13 are provided as a Source Data file.

## Source Data

Source Data
