# Supplementary material for: 245 MHz bandwidth organic light-emitting diodes used in a gigabit optical wireless data link
Source: Nat Commun. 2020 Mar 3;11:1171. doi: 10.1038/s41467-020-14880-2 (PMC7054290; doi:10.1038/s41467-020-14880-2)
Supplement: Supplementary file 1 — Supplementary information [file 41467_2020_14880_MOESM1_ESM.pdf]

Supplementary Information

245 MHz bandwidth organic light-emitting diodes used in a gigabit optical wireless data link

Kou Yoshida[1)], Pavlos P. Manousiadis[1)], Rui Bian[2)], Zhe Chen[2)], Caroline Murawski[1)], Malte C. Gather[1)], Harald Haas[2),a)], Graham A. Turnbull[1),a)], and Ifor D. W. Samuel[1,a)]

1) Organic Semiconductor Centre, SUPA, School of Physics and Astronomy, University of St Andrews, St Andrews KY16 9SS, UK.
2) Li-Fi R&D Centre, Institute for Digital Communications, University of Edinburgh, Edinburgh EH9 3JL, UK.

a) Authors to whom correspondence should be addressed. Electronic mail: Harald.Haas@ed.ac.uk; gat@st-andrews.ac.uk; idws@st-andrews.ac.uk

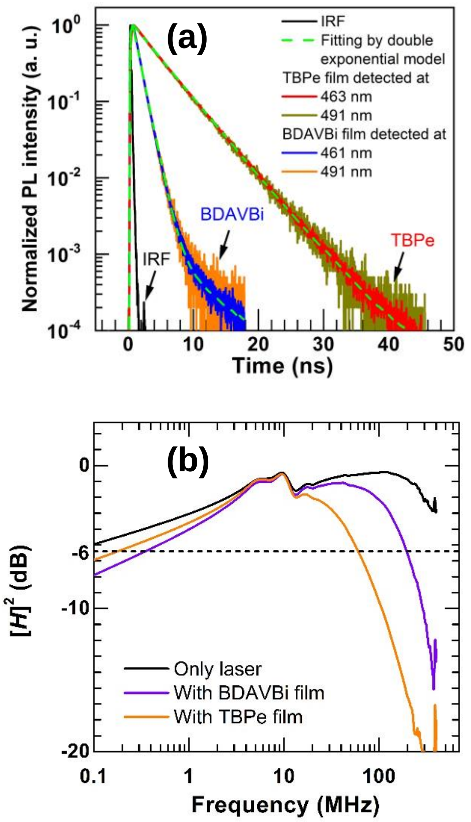

Supplementary Figure 1. (**a**) Transient PL decay curves of films of TBPe and BDAVBi detected at different emission wavelengths (coloured solid lines). The black solid line is the instrument response function (IRF). The green dashed lines are fits by a two-exponential decay. The detection wavelength does not affect the decay curve. (**b**) Frequency response of the films and comparison with the frequency response of the system, i.e., without the films.

Supplementary Note 1: Performance of OLEDs

First generation OLED

Supplementary Figure 2a displays the current density and radiance as a function of voltage for the G1-OLEDs. These reached external electroluminescence (EL) quantum efficiency (EQE) of 3.7% at 100 mA/cm^2, which is typical for OLEDs based on fluorescent emitters. We observed blue emission with a peak emission wavelength of 463 nm, similar to the fluorescence spectra of the TBPe films and TBPe in polymer films[1] (**Supplementary Figure 2b**).

Second generation OLEDs

The G2-OLEDs achieved much higher current density and radiance than the G1-OLEDs (see **Supplementary Figure 2a**). This improvement is due to the high thermal conductivity of the silicon substrate[2,3] and the reduced voltage drop in the silver contact wiring. The similar current densities for devices of different size at voltages higher than 3.0 V indicates that the G2-OLED size does not significantly affect its electrical characteristics. Their spectra are shown in **Supplementary Figure 2b**.

Third generation OLEDs

Supplementary Figure 2a shows current density and radiance of the G3- and G3-FEM-OLEDs as a function of voltage. For a given voltage the current density for G3- and G3-FEM-OLEDs was similar to each other showing that the use of BDAVBi in the G3-FEM-OLEDs did not significantly change charge transport through the OLED. We attribute the slightly lower current than for G2-S-OLEDs to batch to batch variations in the Cs doping concentration in the ETL.

It was observed that the G3-FEM-OLED had higher radiance than the G3-OLED. This cannot be explained by the differences in charge carrier balance or difference emission efficiency of the emitters, since the current density-voltage characteristics are similar for the G3- and G3-FEM-OLED (see **Supplementary Figure 2a**) and higher PL quantum yields for the TBPe films than the BDAVBi doped MADN films (79% for the BDAVBi films and 88% for the TBPe films), and was instead attributed to a better out-coupling efficiency. BDAVBi was reported to show horizontal orientation in a 4,4′-bis(N-carbazolyl)-1,1′-biphenyl matrix, and this is the preferred orientation for higher out coupling efficiency[4]. This also explains the higher current efficiency of the G3-FEM-OLED in **Supplementary Figure 2c**.

Supplementary Figure 2b shows EL spectra of the G3- and G3-FEM-OLEDs and the PL spectra of the corresponding films of the emission layers. We attribute the small difference between the spectra of devices and the corresponding emissive layer to microcavity effects in the OLEDs.

The significant reduction in current efficiency of the OLEDs at higher voltage (**Supplementary Figure 2c**) is attributed to a reduction of emission lifetime due to non-radiative processes such as exciton- exciton and exciton-polaron interactions.

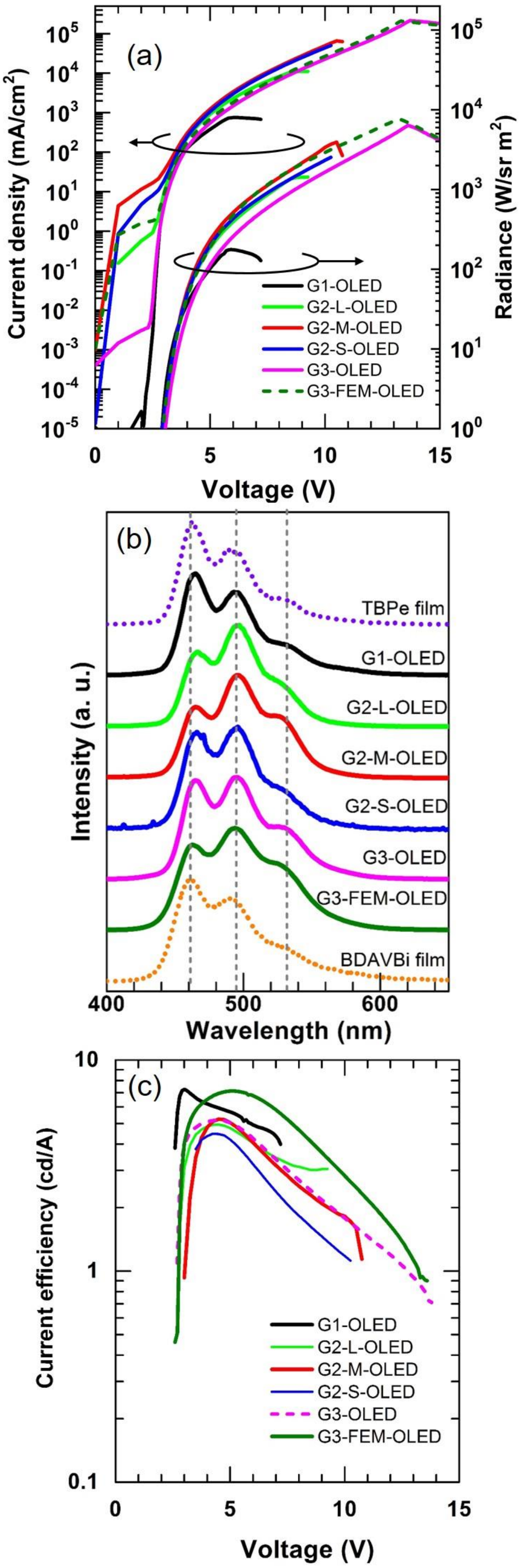

Supplementary Figure 2 (**a**) Current density and light output of OLEDs as a function of voltage. (**b**) EL spectra of OLEDs and comparison with PL spectra of the films. (**c**) Current efficiency of the OLEDs as a function of voltage.

Supplementary Note 2: Enhancement of bandwidth by reduction of device resistance

A simplified equivalent electrical model of OLEDs can be written as a single parallel capacitor (C_d) and resistor (R_d) network with a series resistor (R_s) as shown in **Supplementary Figure 3a**. C_d is a capacitor equivalent to the dielectric behaviour of the organic layers sandwiched by the metals and the junction capacitance induced by the energy barrier between the different layers. R_d represents the resistance of organic layers and R_s represents the contact resistance and wiring resistance of the electrodes. At low frequency, C_d results into open circuit. The sum of R_s and R_d can be estimated as a reciprocal of the slope of the current-voltage characteristics of OLEDs, i.e., differential resistance. R_s is due to transparent electrode, so it is expected not to vary with voltage, and the change in differential resistance with voltage can be attributed to change of R_d. **Supplementary Figure 3b** shows the measured differential resistance of OLEDs as a function of voltage. In **Supplementary Figure 3b**, reduction of R_s as voltage increases, is observed. This is a typical behaviour of diodes, since they have nonlinear current-voltage characteristics.

The frequency response of the OLED is a result of many different processes taking place, including charge transport and emission processes. Here, we are focus on the effect of R_d, with the assumption that other processes are fast enough to ignore them. In this scenario, the effect of R_d on frequency response of OLED can be estimated from the transfer function of the electrical mode shown in **Supplementary Figure 3a**. As EL emission is produced by recombination of charge carriers, it is proportional to the current passing through R_d, and so its emission intensity can be estimated from the voltage across R_d. The frequency response of OLED can be estimated from the frequency transfer function of the electrical model. The voltage gain of the frequency transfer function of the model can be expressed as:

$$|H(j\omega)| = \frac{1}{\sqrt{\left(1+\frac{R_s}{R_d}\right)^2 + (\omega C_d R_s)^2}},$$

where $j^2=-1$ and ω is angular frequency. The 3dB bandwidth of the transfer function (f_{3dB}) can be expressed as:

$$f_{3\mathrm{dB}} = \frac{1}{2\pi C_\mathrm{d}}\left\{\frac{1}{R_\mathrm{s}} + \frac{1}{R_\mathrm{d}}\right\}.$$

Thus, f_{3dB} increases with reduction of C_d, R_s, or R_d. Importantly, R_d is usually larger than R_s ($R_d >> R_s$), especially at low voltage. In this case, f_{3dB} only depends on C_d and R_s and it is the same as the 3dB bandwidth of simple low-pass RC filter, $f_{RC}=1/(2\pi R_s C_d)$. With increasing of voltage, R_d will decrease with resulting increase of f_{3dB}. This phenomenon can be quantitatively simulated as follows. Enhancement of f_{3dB} can be expressed as:

$$\frac{f_{3dB}}{f_{3dB,V=0}} = 1 + \frac{R_s}{R_d}.$$

Supplementary Figure 3a shows the enhancement as a function of R_s/R_d. At $R_d \gg R_s$, there is not a significant improvement of f_{3dB}. The enhancement become significand when R_d reduces to be comparable with R_s, and increases rapidly when R_d reduces further, i.e. for high voltages.

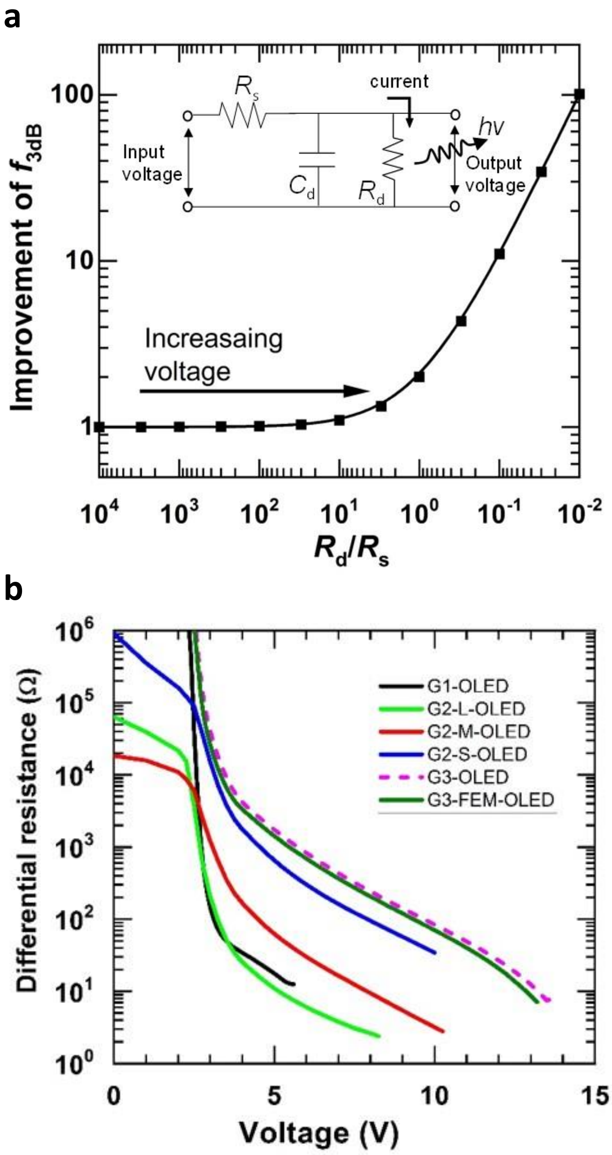

Supplementary Figure 3. (**a**) Enhancement of f_{3dB} as a function of a ratio R_d to R_s. The inset shows the simplified electrical model for OLEDs used for the calculation of enhancement. (b) Differential resistance of OLEDs as a function of voltage.

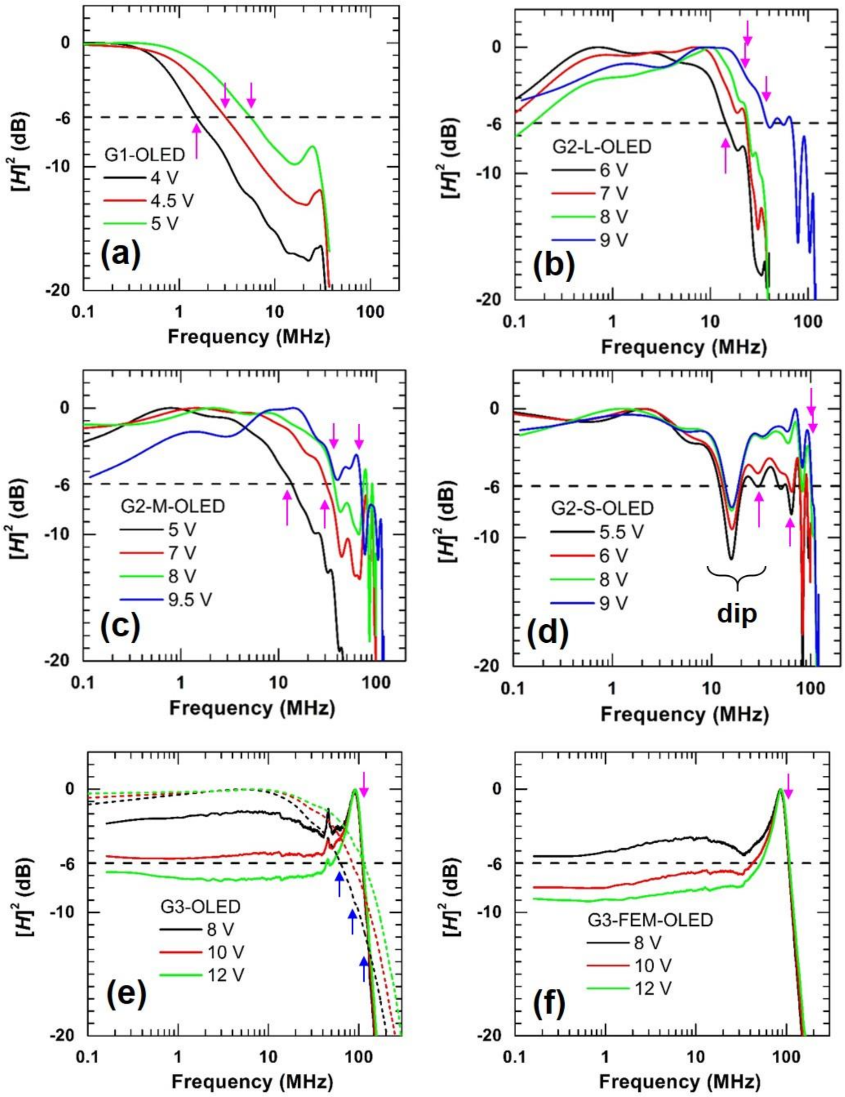

Supplementary Figure 4. Frequency response of the OLEDs at different DC voltage in the VLC link ((**a**) G1-OLED, (**b**) G2-L-OLED, (**c**) G2-M-OLED, (**d**) G2-S-OLED, (**e**) G3-OLED, and (**f**) G3-FEM-OLED). The purple arrows indicate the bandwidth. Here we note that in part (**d**), we ignore the "dip" that appears in the frequency response of the G2-S-OLED to estimate the bandwidth because the signal recovers at higher frequency. Part (**e**) depicts with dashed lines the measured frequency response of the G3-OLED with the photodiode with broader frequency response where the blue arrows indicate the bandwidth at different voltages. Continuous line shows the frequency response of the VLC link, at corresponding voltages. In part (**e**), no change of the bandwidth with voltage can be seen in the VLC link but the bandwidth of OLEDs measured with the photodiode with broader frequency response as a receiver increases with voltage. This is because the custom-made receiver affects the frequency response of the VLC link.

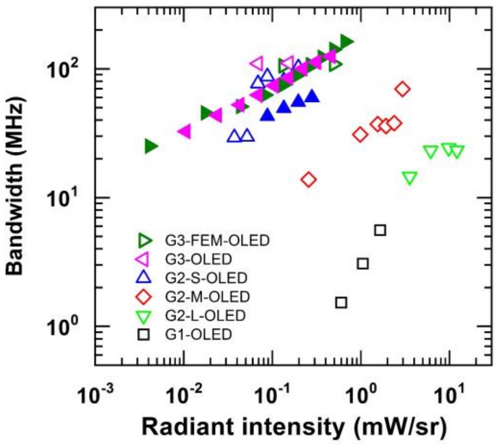

Supplementary Figure 5. bandwidth of OLEDs as a function of radiant intensity. Open symbols represent results in VLC link and solid symbols for OLED.

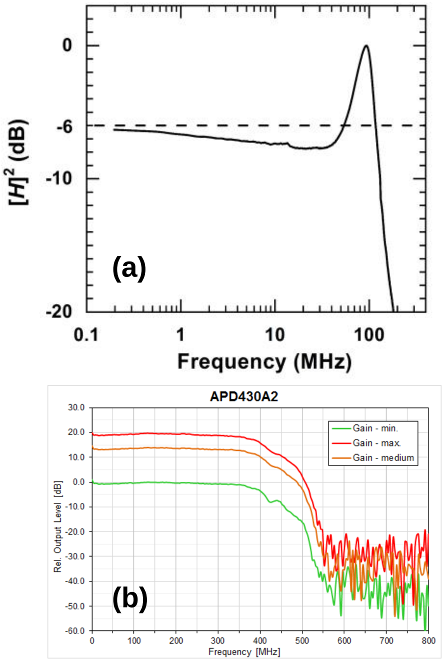

Supplementary Figure 6. (**a**) Frequency response with the custom-made receiver and a laser (Thorlabs, HL6544FM). (b) Reported frequency response of the flatter photodiode (Thorlabs, APD430A2/M). Reprinted from Thorlabs Inc. with permission. Part (**a**) shows that a peak at around 100 MHz in **Supplementary Figure 4e** is due to the resonance peak of the custom-made receiver. Part (**b**) shows that the flatter photodiode has flatter frequency response up to 400 MHz, so we can reliably measure the frequency and the bandwidth of OLEDs up to 400 MHz

Supplementary Note 3: Details of data transmission optimization

To achieve the highest data rate in the OLED-based set-up, we used orthogonal frequency division multiplexing (OFDM), which is an efficient modulation technique. For optical communication based on intensity modulation and direct detection (IM/DD), a modified version of OFDM, such as direct current-biased optical OFDM (DCO-OFDM), should be used. Moreover, due to the low-pass frequency response of the overall channel, adaptive bit loading is applied to optimise the modulation depth at each subcarrier at a target bit error rate (BER). OLEDs are generally nonlinear components (see **Supplementary Figure 2a**), and thus, a vital factor in this system is the DC operation point (i.e., DC bias) of OLEDs. This determines how much the distortion caused by nonlinearity and clipping affects the system performance. In the following, the details of the modulation technique are presented.

DCO-OFDM

OFDM has been a powerful modulation scheme in radio frequency (RF) that enables high spectral efficiency and tackles challenges in RF with simple solutions[5]. However, modifications are necessary to adapt the original OFDM to an optical communication system that uses IM/DD. DCO-OFDM is chosen here because of its efficiency and simple implementation[6]. An OFDM signal is generally complex and bipolar in an RF communication system. However, since the data is carried by the intensity of light in the OLED-based system, the electrical signal fed into the OLED must be real and positive. This can be ensured by first imposing Hermitian symmetry on the subcarrier domain data vector. Then, a constant DC bias value is added to the resultant bipolar signal in the time domain (i.e., after inverse Fourier transform). Assuming there is a large enough DC bias value, which is the case in this paper, it can be ensured that most of the time domain signal is positive. Assuming N available subcarriers and the subcarrier data vector $\boldsymbol{X} = [X_k]_{k=0}^{N-1}$, only $N/2 - 1$ subcarriers are used for data encoding because two subcarriers are reserved for DC bias $X_0 = X_{N/2} = 0$, and Hermitian symmetry is ensured by $X_k = X_{N-k}^*$. Required operations are applied on the subcarrier vector, including inverse fast Fourier transform (IFFT) and addition of cyclic prefix. The generated time domain signal is then sent to the arbitrary waveform generator (AWG). The dynamic range of the AWG in our experiment is -1 V to +1 V. Therefore, extreme values of the signal generated by the computer are also clipped, ±3.2 V in our experiment, to increase the signal resolution after scaling to the dynamic range of the AWG and minimizing the signal distortion.

Adaptive bit loading

The overall frequency response of the system is affected by both optical and electrical components. In this paper, the frequency response of the channel is mostly limited by the OLEDs and is characterised as a lowpass response as shown in **Supplementary Figure 4**. In other words, each subcarrier experiences a different value of signal-to-noise ratio (SNR). Assuming that the binary data is modulated by a M_k-ary quadrature amplitude modulation (M_k-QAM) format at kth subcarrier, the maximum possible modulation depth M_k can be determined at each subcarrier based on the effective SNR at that subcarrier, SNR_k, and the target BER[7]. The BER at kth subcarrier with the effective SNR, SNR_k, is approximated by[8]

$$\mathrm{BER} \cong \frac{4\left(1-\frac{1}{M_k}\right)}{\log_2 M_k} \sum_{p=1}^{\min(2,\sqrt{M_k})} \mathrm{Q}\left((2p-1)\sqrt{\frac{3\mathrm{SNR}_k \log_2 M_k}{2(M_k-1)}} \right),$$

where $\mathrm{Q}(.)$ is the Gaussian Q-function. SNR values at all subcarriers are estimated using a known training sequence (e.g., 4-QAM modulated OFDM signal) prior to data transmission. Then, based on the estimated SNR and the target BER of 3.8×10^{-3} the maximum modulation depth at each subcarrier is calculated using the equation above. Therefore, the total data rate is estimated as[9]

$$R = \frac{\sum_{k=1}^{N} \log_2 M_k}{2B(N + N_C)},$$

where B is the single-sided modulation bandwidth of the system, and N_C is the number cyclic prefix. Note that random data are then transmitted in 35 independent OFDM frames, with 1024 subcarriers each, according to the designated modulation depths, and the actual BER rate is measured to ensure that the total BER is below the target. The estimated SNR and the corresponding bit loading for the highest achieved data rate are presented in **Fig. 4**.

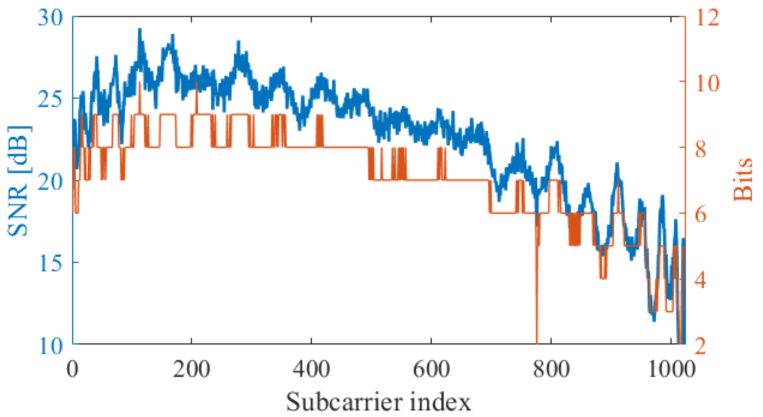

Supplementary Figure 7. The estimated SNR and bit loading results for the fastest G3-FEM-OLED with data rate 1.17 Gbps and BER of 3×10^{-3}.

The effect of bias point and scaling factor

It is well established that LEDs are nonlinear media, in the sense that the relation between their input DC voltage and output optical intensity is not linear[10]. As mentioned above and shown in **Supplementary Figure 2a**, this is also the case for OLEDs. Therefore, an additional distortion source, which reduces the effective SNR, is added to the communication system. The effect of nonlinearity can be alleviated by selecting an optimum operation point (i.e., DC bias voltage) in the linear OLED operation regime. Also, the signal can be scaled, by setting a signal peak-to-peak voltage V_{pp}, so that it fits in the most linear part of the voltage-intensity curve. Therefore, the highest data rate is obtained by finding the optimum bias voltage and scaling the signal (i.e., choosing V_{pp}). This ensures that the effect of nonlinearity is minimized. Data rate results for various DC voltages are presented in **Figure 2b** of the main text. For each result, various V_{pp} values were tested and the one that led to the highest data rate was selected.

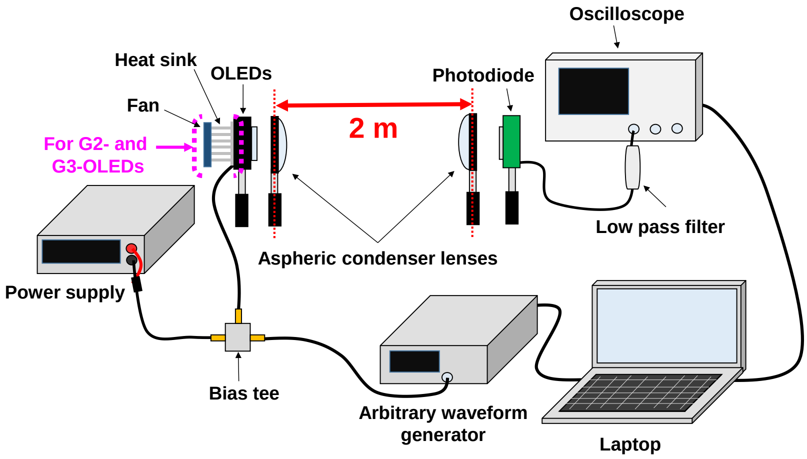

Supplementary Figure 8. Schematic illustration of the VLC measurement system used in this study.

(a)

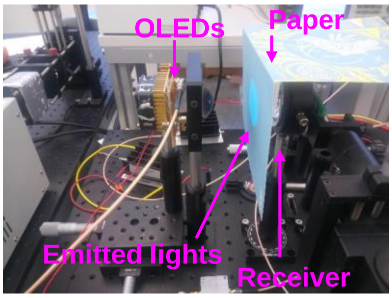

(b)

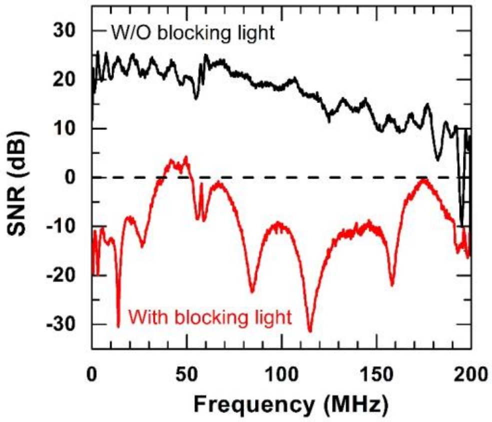

Supplementary Figure 9. (**a**) Photograph of the experiment to confirm that the data transmission was due to the light emitted by the G3-FEM-OLED. (**b**) SNR spectra of the OLED in 5 cm data link at a fixed voltage of 12 V without (black line) and with paper inserted to block the optical link (red line). The SNR drops to almost 0 dB when the light is blocked, showing that the data transmission is due to the light emitted by the G3-FEM-OLED.

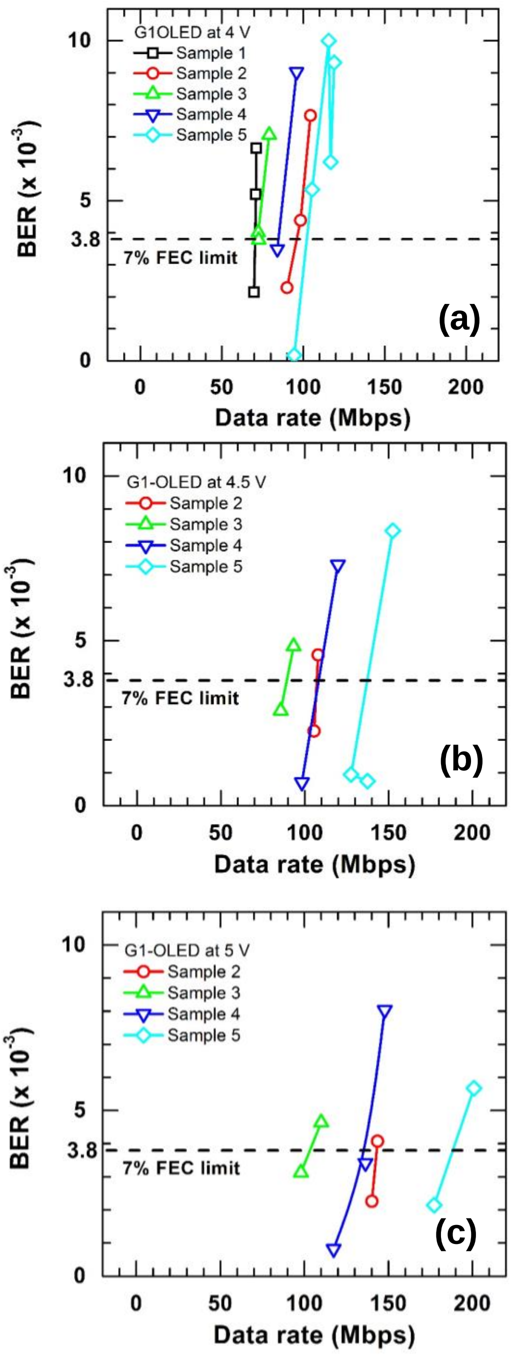

Supplementary Figure 10. BER as a function of data rate of the G1-OLEDs in 2 m data link at different voltages and for different OLED samples: (**a**) at V_{DC} = 4 V, (**b**) at V_{DC} = 4.5 V, and (**c**) at V_{DC} = 5.0 V.

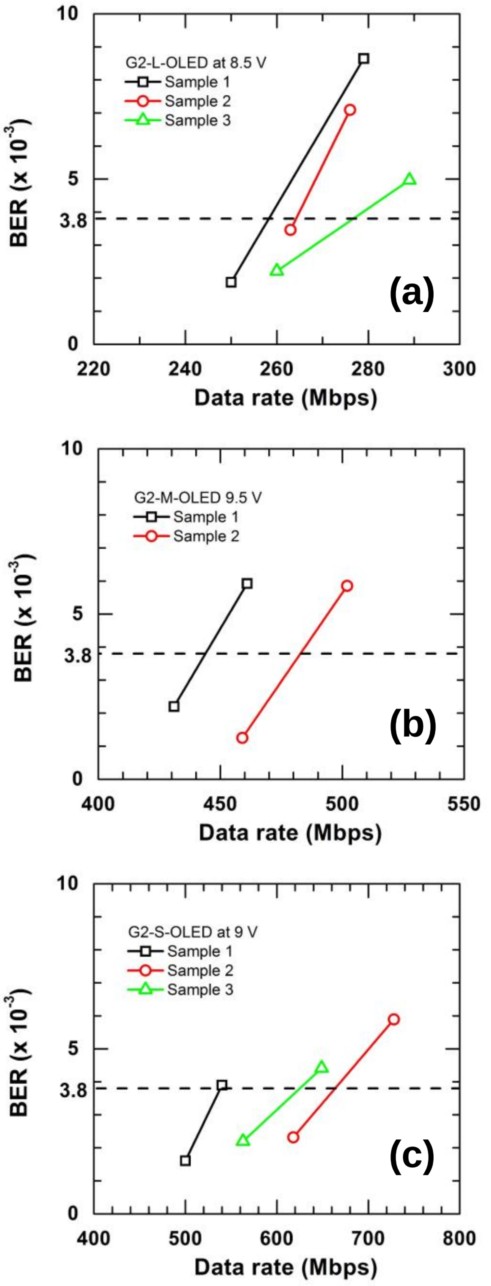

Supplementary Figure 11. BER as a function of data rate of the G2-OLEDs for different OLED samples in a 2 m data link: (**a**) G2-L-OLED at V_{DC} = 8.5 V, (**b**) G2-M-OLED at V_{DC} = 9.5 V, and (**c**) G2-S-OLED at V_{DC} = 9 V.

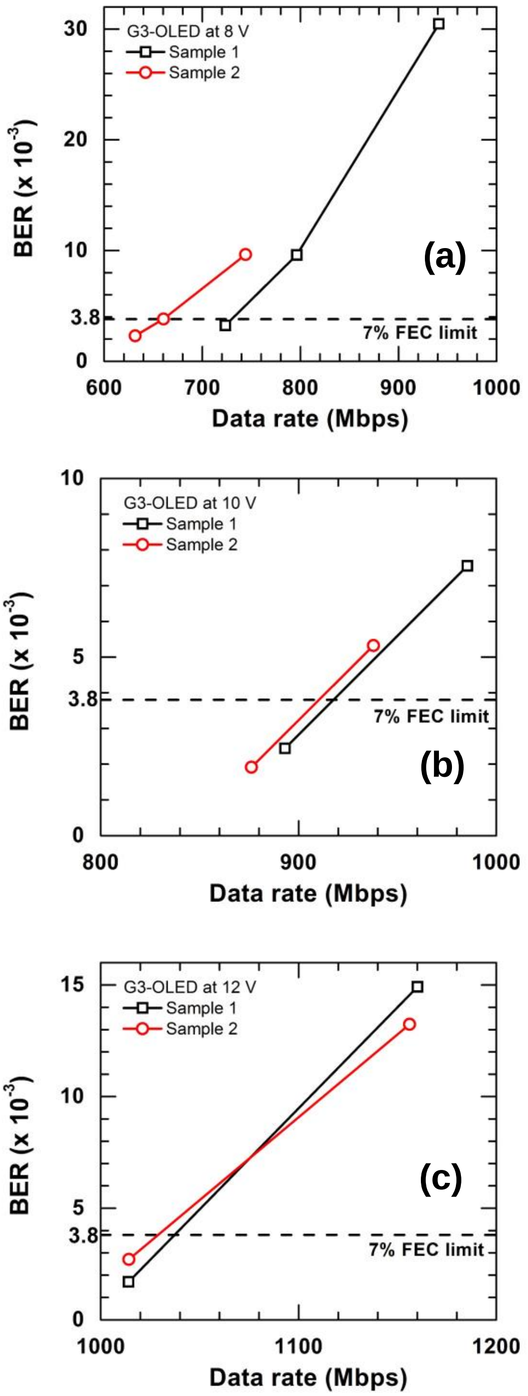

Supplementary Figure 12. BER as a function of data rate of the G3-OLEDs in a 2 m data link at 3 different voltages for 2 OLED samples: (**a**) at V_{DC} = 8 V, (**b**) at V_{DC} = 10 V, and (**c**) at V_{DC} = 12 V.

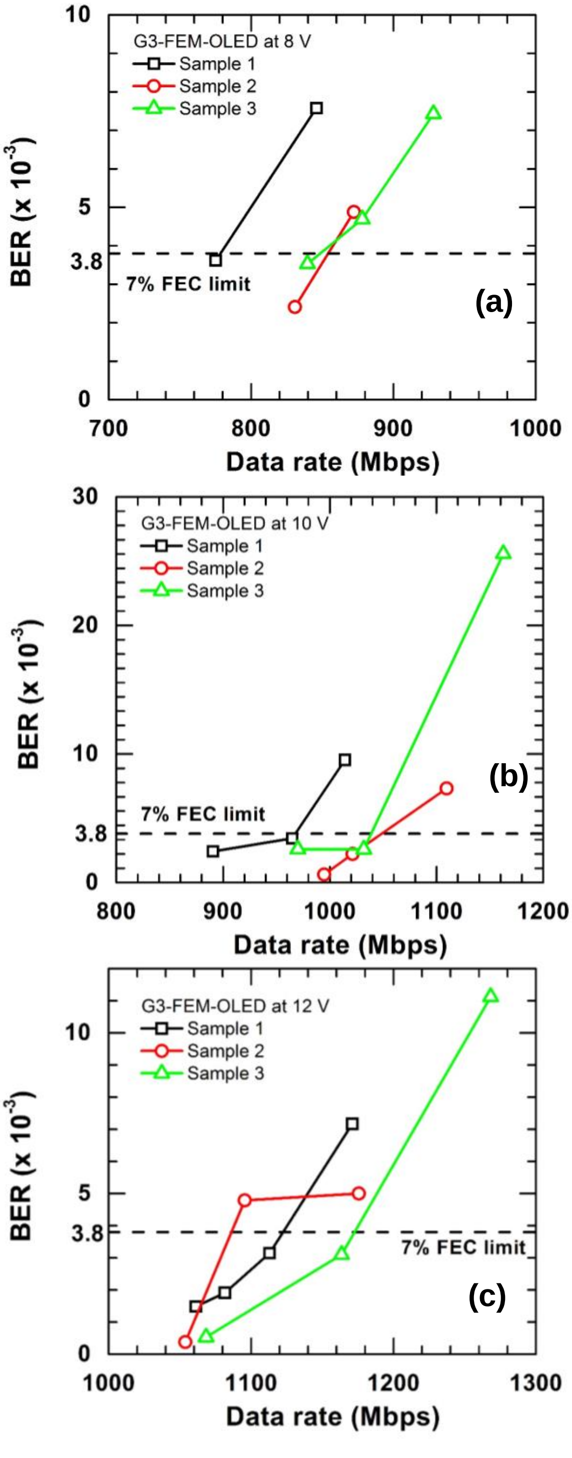

Supplementary Figure 13. BER as a function of data rate of the G3-FEM-OLEDs for different samples at same operation condition: (**a**) at V_{DC} = 8 V in a 2 m data link, (**b**) at V_{DC} = 10 V in a 2 m data link, and (**c**) at V_{DC} = 12 V in a 2 m data link.

Supplementary Table 1. Summary of date rate measurements for each generation of OLED in a 2m link. The table shows the effect of operating voltage and also the data rate measured for each of several OLEDs of each type.

Devices	Voltage [V]	Number of samples	Data rate of each OLED sample [Mbps]	Averaged data rate [Mbps]
G1-OLED	4	5	70.8, 72.7, 84.9, 96.7, 102	90
	4.5	4	89.3, 107, 109, 137	110
	5	4	103, 136, 143, 188	140
G2-L-OLED	8.5	3	258, 264, 277	270
G2-M-OLED	9.5	2	444, 483	460
G2-S-OLED	9	3	538,625,663	610
G3-OLED	8	2	663, 733	700
	10	2	910, 917	913
	12	2	1029, 1038	1033
G3-FEM-OLED	8	3	778, 849, 855	820
	10	3	968,1038, 1049	1020
	12	3	1086, 1124, 1171	1130

Supplementary References

1 Verhagen, J. C. D., van Zandvoort, M. A. M. J., Vroom, J. M., Johansson, L. B. Å. & van Ginkel, G. Spectroscopic Properties of 2,5,8,11-Tetra-tert-butylperylene in Polymer Films. *The Journal of Physical Chemistry B* **101**, 10568-10575, doi:10.1021/jp972179e (1997).

2 Yamamoto, H., Kasajima, H., Yokoyama, W., Sasabe, H. & Adachi, C. Extremely-high-density carrier injection and transport over 12000A⁄cm2 into organic thin films. *Applied Physics Letters* **86**, 083502, doi:10.1063/1.1866230 (2005).

3 Chung, S., Lee, J.-H., Jeong, J., Kim, J.-J. & Hong, Y. Substrate thermal conductivity effect on heat dissipation and lifetime improvement of organic light-emitting diodes. *Applied Physics Letters* **94**, 253302, doi:10.1063/1.3154557 (2009).

4 Frischeisen, J., Yokoyama, D., Endo, A., Adachi, C. & Brütting, W. Increased light outcoupling efficiency in dye-doped small molecule organic light-emitting diodes with horizontally oriented emitters. *Organic Electronics* **12**, 809-817, doi:10.1016/j.orgel.2011.02.005 (2011).

5 Weinstein, S. B. The history of orthogonal frequency-division multiplexing [History of Communications]. *IEEE Communications Magazine* **47**, 26-35, doi:10.1109/MCOM.2009.5307460 (2009).

6 Haas, H., Yin, L., Wang, Y. & Chen, C. What is LiFi? *J. Lightwave Technol.* **34**, 1533-1544, doi:10.1109/JLT.2015.2510021 (2016).

7 Levin, H. E. in *GLOBECOM'01. IEEE Global Telecommunications Conference (Cat. No.01CH37270).* 369-374 vol.361.

8 Tsonev, D., Videv, S. & Haas, H. Unlocking Spectral Efficiency in Intensity Modulation and Direct Detection Systems. *IEEE Journal on Selected Areas in Communications* **33**, 1758-1770, doi:10.1109/JSAC.2015.2432530 (2015).

9 Hahn, T. *et al.* Monomolecular and Bimolecular Recombination of Electron–Hole Pairs at the Interface of a Bilayer Organic Solar Cell. *Advanced Functional Materials* **27**, 1604906, doi:doi:10.1002/adfm.201604906 (2017).

10 Tsonev, D., Sinanovic, S. & Haas, H. Complete Modeling of Nonlinear Distortion in OFDM-Based Optical Wireless Communication. *Journal of Lightwave Technology* **31**, 3064-3076, doi:10.1109/JLT.2013.2278675 (2013).
